# Understanding the Clinical Features of Coronavirus Disease 2019 From the Perspective of Aging: A Systematic Review and Meta-Analysis

**DOI:** 10.3389/fendo.2020.557333

**Published:** 2020-11-11

**Authors:** Chen Yifan, Pu Jun

**Affiliations:** State Key Laboratory for Oncogenes and Related Genes, Division of Cardiology, Renji Hospital, Shanghai Cancer Institute, Shanghai Jiao Tong University School of Medicine, Shanghai, China

**Keywords:** 2019 novel coronavirus, 2019 coronavirus disease, aging patients, meta-analysis, immune senescence, clinical characteristics

## Abstract

**Problems:**

An outbreak of novel coronavirus (2019-nCov) infection is now widespread in multiple countries. Compared with adult patients, elderly patients have not received enough attention. The aim of the meta-analysis was to assess the clinical characteristics of elder patients with COVID-19.

**Methods:**

A deep literature search was performed in the databases through August 21, 2020. Risk ratio (OR) and 95% confidence intervals (CIs) were pooled using analysis models.

**Results:**

Three studies including 2046 infected patients were precisely evaluated, and the results show that the elderly group has a higher risk of hypertension, diabetes, and cardiovascular disease than the younger patients. Their total white blood cells are higher than that of the younger patients, and their lymphocytes are relatively reduced compared with the younger patients.

**Conclusion:**

We comprehensively assessed the clinical characteristics of patients of different ages with COVID-19 and found that elder patients had a high risk of chronic cardiovascular and metabolism comorbidities. The characteristic clinical manifestations and laboratory examinations of elderly patients support their excessive inflammation and weak immune defenses against 2019-nCoV. All these findings provide important information for understanding the general clinical characterization of the aging immune defense against the virus and enhancing the public awareness of the prevention and treatment of elder patients in the COVID-19 pandemic.

## Introduction

Since a group of unexplained pneumonia related to the Huanan seafood market was reported in Wuhan, China, on December 31, 2019 (1), this new coronavirus, named 2019 novel coronavirus (2019-nCoV) or severe acute respiratory syndrome coronavirus 2 (SARS-CoV-2), which was soon confirmed by Chinese scientists as a new betacoronavirus (2), is now causing the 2019 coronavirus disease (COVID-19) pandemic. It has been undoubtedly confirmed that the number of incidence cases, the total death toll, the duration of the epidemic, and the property damage to the international community caused by this virus pandemic are far superior to other coronavirus infectious diseases, including the severe acute respiratory syndrome (SARS) caused by the coronavirus (SARS-CoV) from 2002 to 2003 (3) and the middle east respiratory syndrome (MERS) caused by the coronavirus (MERS-CoV) since 2012 (4). Although the global fatality rate of this disease has yet to be determined, it has caused more than 22,054,300 infections and 779,443 deaths worldwide until August 19, 2020 (5), and has brought huge short- and long-term losses to the globe economy without specific therapies.

It is a huge challenge for the global health system, especially for developed communities with a high proportion of elderly people. Interpersonal transmission of this new virus has even occurred in many nursing facilities around the world (6). In past research, aging is considered to be a complex multilevel change, characterized by a progressive physiological dysfunction and disability to stabilize the homeostasis, leading to an increased incidence of degenerative diseases and deaths (7). At the same time, aging shows significant modulations at the cellular and system level, including the formation of senescent cells and the imbalance of cytokine regulation. Among them, the most obvious phenomenon is the disorder of the immune system, including immune decline caused by changes in the number and proportion of immune cells and chronic inflammation caused by high expression of inflammatory factors, which was also called “inflammaging” or “immune senescence” (8). The decline of the immune system weakens the immune function of the elderly population against pathogens such as the coronavirus, and the state of chronic inflammation increases the risk of cytokine storms.

Although some information about the epidemiology of older patients with COVID-19 has been accumulated, there is a lack of relevant comprehensive reports, and the related protective and diagnostic measures are not perfect. Therefore, it is urgent to pay attention to the prevention and control of infectious diseases in elderly people in aging society today because people of different ages are generally susceptible to this virus. Meanwhile, comparing the differences of clinical features between two patients of different ages can help doctors to carry out targeted individual treatments all over the world to save unnecessary waste of medical resources and maximize medical efficiency. Here, we have systematically reviewed the single-center or multicenter observational studies of older patients with COVID-19 and comprehensively dissected the true impact of age as a complex variable on COVID-19 disease.

## Methods

### Search Strategy and Selection Criteria

A literature search was performed on studies published from December 1, 2019, to August 21, 2020, in PubMed, EMBASE, Cochrane Library, Scopus, and Web of Science databases without restriction to regions or languages. The following terms—”Coronavirus” OR “Coronavirus infections” OR “betacoronavirus 1” OR “betacoronavirus” OR “SARS-CoV-2” OR “COVID-19” OR “2019-nCoV” OR “new coronary pneumonia” OR “novel coronavirus” OR “coronavirus disease” OR “coronavirus disease 2019” OR “2019 novel coronavirus infection” combined with “descriptive study” OR “retrospective study” OR “cross-sectional study” OR “case-control study” OR “cohort study”—were used for searching. We also searched the preprint platform medRxiv, bioRxiv, and the reference list of each selected study to make sure not to miss relevant papers. All retrieved publications were managed by EndNote X9.0 software.

The studies were selected based on the Strengthening the Reporting of Observational Studies in Epidemiology (STROBE) Statement. Eligibility criteria are as follows: (a) research types: descriptive studies including case-control studies, retrospective cross-sectional studies, cohort studies, and case series; (b) research subjects: patients with laboratory-confirmed COVID-19; (c) studies comparing clinical characteristics, laboratory findings, and outcomes for the elderly and young. Exclusive criteria are as follows: (a) study types: reviews, editorials, opinions, letters, case reports, consensus documents, meta-analyses, and clinical trials; (b) studies, including duplicate data; (c) studies about nonhuman SARS-CoV-2 infection; (d) studies without young patients as controls.

### Data Collection and Quality Assessment

Two independent investigators reviewed titles and abstracts of all retrieved studies to include eligible studies and then extracted data from these proper studies using a predefined data extraction form. Disagreements in the process between two investigators were resolved by discussion or consensus with a third investigator. Extracted data included the following: study name, first author name, date of publication, country, sample size, baseline features (i.e., mean age, sex, clinical symptoms, comorbidities), lung computed tomography (CT), therapies, complications and prognosis. The risk of bias of selected studies was assessed using the Newcastle-Ottawa Scale (NOS) standard, which consists of three factors: patient selection, comparability of the study groups, and assessment of outcome. A score of <6 means a low-quality study and would be excluded from meta-analysis.

### Statistical Analysis

Categorical variables were reported as the number of cases and percentage, and continuous variables were reported as mean ± standard deviation (SD). We used the data conversion method proposed to estimate mean and standard deviation when the studies only include median and interquartile range (IQR) (9). All statistical analysis was performed by STATA MP, version 16.0. For meta-analysis, the standard mean difference (SMD) and odds ratio (OR) were respectively used to compare continuous and categorical outcomes. All results were reported with 95% confidence intervals (CIs), and were presented as Forest plots. I-square (I^2^) value was used to evaluate heterogeneity across studies, with I^2^ ≤ 50% representing statistically homogeneous and I^2^>50% representing statistically heterogeneous. If I^2^ is negative, we set it to 0.00%. We performed a fixed-effects model using the Mantel Haenszel statistical method to evaluate average effects when I^2^ ≤ 50% and a random-effects model with REML method when I^2^>50%. The significance level of the meta-analysis was set to α = 0.05. Because the number of included studies is small, we didn’t assess the publication bias by both funnel plots and Egger’s linear regression test.

## Results

### Research Flow and the Baseline Data of the Aging Patients With COVID-19

**Figure 1:**
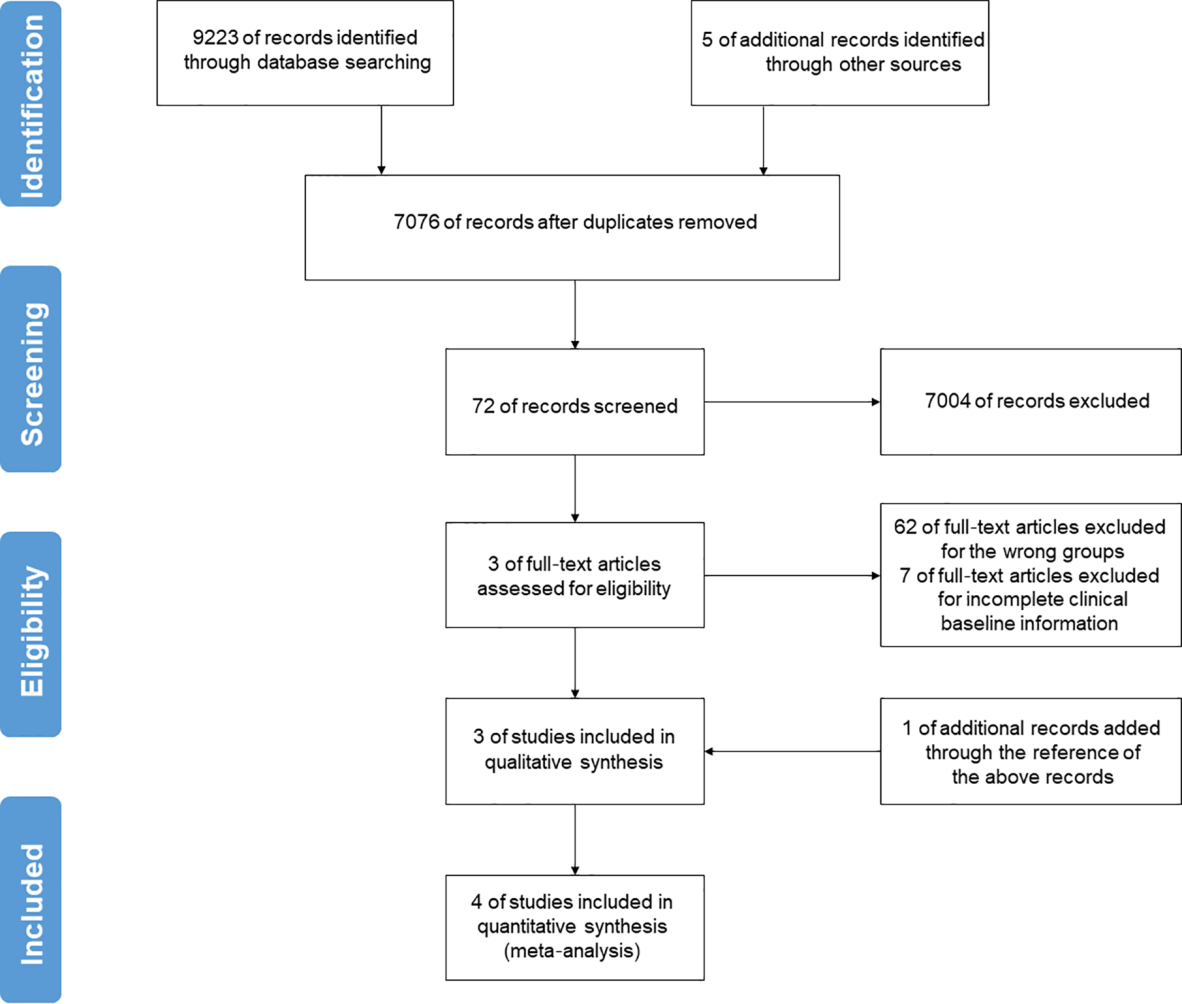
Flow-chart of the literature search and selection strategy in this meta-analysis. After applying the inclusion and exclusion criteria, a total of 4 studies were included in the final pooled analysis.

A preliminary search included a total of 9228 publications. After removing duplicates, a total number of 7076 documents could be initially identified. By overlapping articles and excluding case reports, reviews, editorials, opinions, and randomized controlled trials (RCTs) by the title and abstract, 7004 documents were left. Then, 62 studies were excluded because they were not designed to group by age, and 7 studies were excluded because they provided incomplete clinical baseline data after reviewing the full text. One additional study was included from the reference list of included articles. Therefore, four descriptive researches were included in the final pooled analysis (**Figure 1**), meeting the predetermined inclusion and exclusion criteria with a sample of 2047 patients confirmed with COVID-19 (736 aging patients, 35.96% of the whole) (10–13).

As the baseline presented in **Table 1 (A)**, 2047 patients confirmed with COVID-19 from different areas in China were included in the meta-analysis. The average ages of the older group are 67.67 ± 3.62, 68.28 ± 7.31, 76.08 ± 4.73, and 76.67 ± 19.79 years and 12 (66.67%), 58 (42.65%), 250 (47.44%), and 34 (61.81%) of them are men, and the average ages of the younger group are 44.67 ± 11.94, 41.15 ± 11.38, 44.33 ± 14.13, and 43.33 ± 32.94 years and 19 (50.00%), 349 (53.53%), 216 (45.67%), and 74 (50.00%) are men.

Table 1Baseline characteristics and chronic comorbidities of the included studies in this meta-analysis.

**Table:** (A) Baseline characteristics of the aging patients with COVID-19.

Studies	Years	Countries	Provinces	Centers	No. of patients			Age		Sex (Male/Female)		
					Total	Older	Younger	Older	Younger	Total	Older	Younger
Liu et al. (12)	2020	China	Hainan	Single-center	56	18	38	67.67 ± 3.62	44.67 ± 11.94	31/25	12/6	19/19
Lian et al. (10)	2020	China	Zhejiang	Multi-center	788	136	652	68.28 ± 7.31	41.15 ± 11.38	407/381	58/78	349/303
Zhao et al. (13)	2020	China	Hubei	Single-center	1000	527	473	76.08 ± 4.73	44.33 ± 14.13	466/534	250/277	216/257
Chen et al. (11)	2020	China	Hubei	Single-center	203	55	148	76.67 ± 19.79	43.33 ± 32.94	108/95	34/21	74/74

**Table:** (B) Chronic comorbidities of the aging patients with COVID-19.

**Study**	**Hypertension**			**Diabetes**			**Chronic liver disease**			**Chronic renal disease**		
	**Total**	**Younger**	**Older**	**Total**	**Younger**	**Older**	**Total**	**Younger**	**Older**	**Total**	**Younger**	**Older**
**Zhao et al.**	282	62	220	118	34	84	29	12	17	24	7	17
**Liu et al.**	10	5	5	4	1	3	1	0	1	1	1	0
**Chen et al.**	43	22	21	16	4	12	8	6	2	8	5	3
**Lian et al.**	126	73	53	57	33	24	31	25	6	7	5	2
**Study**	**Heart disease**			**COPD**			**Immune dysfunction**			**Cancers**		
	**Total**	**Younger**	**Older**	**Total**	**Younger**	**Older**	**Total**	**Younger**	**Older**	**Total**	**Younger**	**Older**
**Zhao et al.**	60	6	54	23	0	23	13	3	10	28	7	21
**Liu et al.**	3	0	3	NA	NA	NA	NA	NA	NA	NA	NA	NA
**Chen et al.**	16	5	11	8	1	7	6	5	1	7	2	5
**Lian et al.**	11	5	6	3	0	3	1	0	1	6	3	3

### Higher Risk of Cardiovascular and Metabolic Comorbidities of the Aging Patients With COVID-19

**Figure 2:**
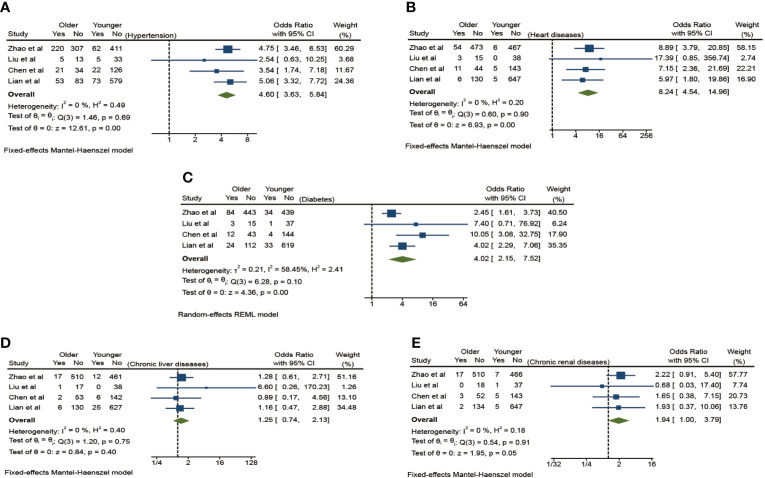
The effect of chronic comorbidities on the risk of elderly patients with COVID-19 compared to younger patients. **(A)** Hypertension, **(B)** Diabetes, **(C)** Heart diseases, **(D)** Chronic liver diseases, **(E)** Chronic renal diseases.

Chronic basic comorbidities of the aging patients with COVID-19 in **Table 1 (B)** include hypertension, diabetes, chronic kidney disease, liver disease, heart diseases, chronic obstructive pulmonary disease (COPD), immune dysfunction, cancers, and other diseases. The result of this meta-analysis shows a significant difference between the elderly group and the young group (**Figure 2**). Compared with the youth group, the elderly group has the higher risk of hypertension, diabetes, and cardiovascular disease (OR 4.60, 95% CI: 3.63–5.84), (OR 4.02, 95% CI: 2.15–7.52), and (OR 8.24, 95% CI: 4.54–14.26) and with a lower heterogeneity (I^2^ = 0.00%) in the fixed-effects models and obvious statistical significance (*p <*0.01) except the analysis of diabetes, which has a slightly larger heterogeneity (I^2^ = 58.45%) in the random-effects model. Although the risk of chronic kidney disease and liver disease is slightly increased (OR 1.94, 95% CI: 1.00–3.79), (OR 1.25, 95% CI: 0.74–2.13), there is no significant statistical difference (*p* > 0.05).

### Differences of Clinical Symptoms in Aging Patients With COVID-19

**Figure 3:**
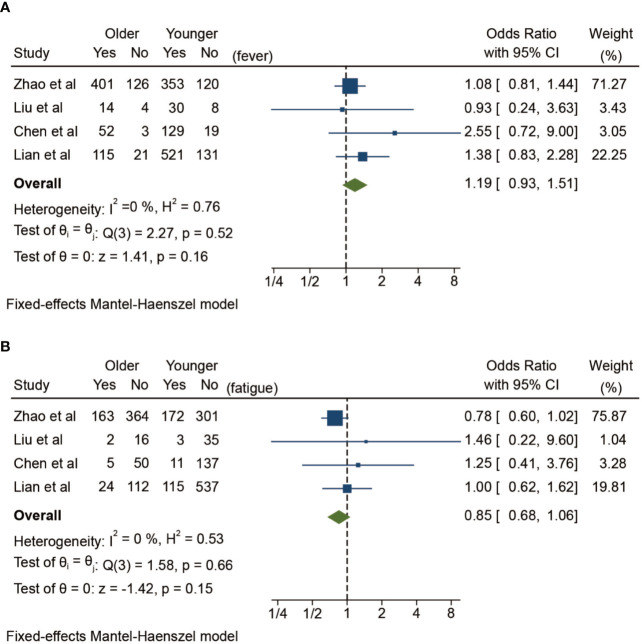
Symptomatic differences between elderly and younger patients with COVID-19 in fixed-effects Mantel-Haenszel model. **(A)** Fever, **(B)** Fatigue.

In addition to comorbidities, the clinical manifestations of elderly patients are also worthy of attention. Here, we have classified and summarized the common clinical manifestations between the two groups in **Table 2** according to general symptoms, respiratory symptoms and extrarespiratory symptoms, which makes it more concise and clearer to show the symptomatic differences. Because different studies have different statistical standards on symptomology, it is difficult to make quantitative analysis of catarrhal symptoms, sore throat, cough, expectoration, chest pain, chest tightness, dyspnea, gastrointestinal symptoms, and neuromuscular symptoms. The meta-analysis results here (**Figure 3**) show that the risk of fever is slightly increased in the elderly group among the common symptoms (OR 1.19, 95% CI: 0.93–1.51), and the risk of fatigue in aging patients is slightly decreased (OR 0.85, 95% CI: 0.68–1.06), but there are no statistically significant differences between the two groups (*p* > 0.05).

Table 2Clinical manifestations of the aging patients with COVID-19 included studies in this meta-analysis.

**Table:** (A) General symptoms and respiratory symptoms of the aging patients with COVID-19.

Studies	General symptoms (Older/Younger)						Respiratory symptoms (Older/Younger)											
	Fever		Fatigue		Catarrhal symptoms		Sore throat		Cough		Expectoration		Chest pain		Chest tightness		Dyspnea	
	Older	Younger	Older	Younger	Older	Younger	Older	Younger	Older	Younger	Older	Younger	Older	Younger	Older	Younger	Older	Younger
**Zhao et al.**	401 (76.09%)	353 (74.63%)	163 (30.93%)	172 (36.36%)	8 (1.52%)	12 (2.54%)	12 (2.28%)	31 (6.55%)	307 (58.25%)	290 (61.31%)	109 (20.68%)	81 (17.12%)	10 (1.90%)	9 (1.90%)	131 (24.86%)	78 (16.49%)	151 (28.65%)	104 (21.99%)
**Liu et al.**	14 (77.78%)	30 (78.95%)	2 (11.11%)	3 (7.89%)	1 (5.56%)	2 (5.26%)	NA	NA	15 (39.47%)/6 (33.33%)				NA	NA	2 (11.11%)/2 (5.26%)			
**Chen et al.**	52 (94.55%)	129 (87.16%)	5 (9.09%)	11 (7.43%)	NA	NA	NA	NA	38 (69.09%)	84 (56.76%)	NA	NA	1 (1.82%)	3 (2.03%)	35 (63.64%)	37 (25.00%)	1 (1.82%)	2 (1.35%)
**Lian et al.**	115 (84.56%)	521 (79.91%)	24 (17.65%)	115 (17.64%)	2 (1.47%)	45 (6.90%)	17 (12.50%)	94 (14.42%)	85 (62.50%)	421 (64.57%)	49 (36.03%)	216 (33.13%)	NA	NA	NA	NA	17 (12.50%)	20 (3.07%)

**Table:** (B) Extra-respiratory symptoms of the aging patients with COVID-19.

Studies	Gastrointestinal symptoms (Older/Younger)										Neuromuscular symptoms (Older/Younger)							
	Anorexia		Nausea		Diarrhea		Abdominal pain		Vomiting		Lethargy		Headache		Dizziness		Muscle ache	
	Older	Younger	Older	Younger	Older	Younger	Older	Younger	Older	Younger	Older	Younger	Older	Younger	Older	Younger	Older	Younger
**Zhao et al.**	73 (13.85%)	61 (12.90%)	13 (2.47%)	8 (1.69%)	52 (9.87%)	48 (10.15%)	4 (0.76%)	3 (0.63%)	15 (2.85%)	11 (2.33%)	4 (0.76%)	6 (1.27%)	13 (2.47%)	19 (4.02%)	18 (3.42%)	15 (3.17%)	24 (4.55%)	42 (8.88%)
**Liu et al.**	NA	NA	NA	NA	NA	NA	NA	NA	3 (16.67%)/7 (18.42%)				NA	NA	NA	NA	NA	NA
**Chen et al.**	5 (9.09%)	1 (0.68%)	1 (1.82%)	2 (1.35%)	7 (12.73%)	3 (2.03%)	3 (5.45%)	1 (0.68%)	1 (1.82%)	2 (1.35%)	NA	NA	3 (5.45%)	7 (4.73%)	1 (1.92%)	3 (2.03%)	11 (20.00%)	43 (29.05%)
**Lian et al.**	11 (8.09%)/77 (11.81%)										NA	NA	8 (5.88%)	67 (10.28%)	NA	NA	20 (14.71%)	71 (10.89%)

### High Inflammatory State and Low Immune Defense of the Aging Patients With COVID-19

**Figure 4:**
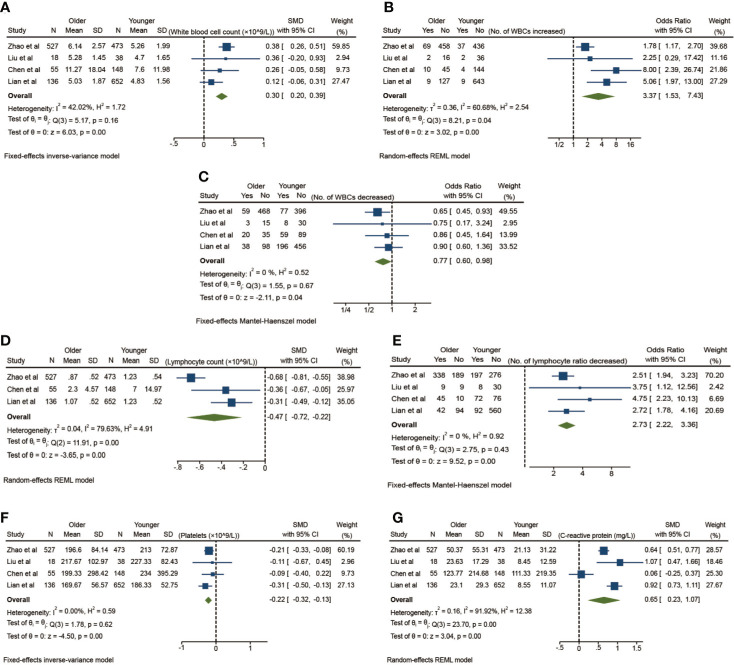
Laboratory tests on the risk of elderly patients with COVID-19 compared to younger patients. **(A)** The total white blood cells, **(B)** Number of increases in the total white blood cells, **(C)** Number of decreases in the total white blood cells, **(D)** Lymphocytes, **(E)** Number of decreases in lymphocytes, **(F)** Platelets, **(G)** C-reactive protein.

Laboratory tests of elderly patients show great heterogeneity according to the differences between the elderly group and the young group in **Table 3**. Here, blood routines are accounted to analyze the changes in the proportion of peripheral blood lymphocytes in elderly patients. Statistical analysis is performed on the C-reactive protein, procalcitonin, and interleukin-6 to infer the inflammatory and immune response of elderly patients, and the platelets and D-dimer are counted to characterize the coagulation function of aging patients. The total number of white blood cells in elderly patients is higher than that in young patients (SMD 0.30, 95% CI: 0.20–0.39, *p* < 0.01) (**Figure 4A**). At the same time, the risk of an increase in the total number of white blood cells in elderly patients is higher than that in the youth group (OR 3.37, 95% CI: 1.53–7.43, *p <*0.01) (**Figure 4B**). Meanwhile, the risk of a decreased white blood cell count in elderly patients was also higher than that in the younger group (OR 0.77, 95% CI: 0.60–0.98, *p* = 0.04) (**Figure 4C**) with absolutely no heterogeneity (I^2^ = 0%). These changes depict a high variability of white blood cells in aging patients, which means a fragile unsteady state caused by COVID-19 in the elderly population.

Table 3Laboratory findings of aging patients with COVID-19 of the included studies in this meta-analysis.

**Table:** (A) Blood routine findings of the aging patients with COVID-19.

Study	White blood cell count (× 10 ^9^/L)		Total white blood cells increased, n (%)		Total white blood cells decreased, n (%)		Lymphocyte count (× 10 ^9^/L)		Lymphocyte ratio, %		Lymphocyte ratio decreased, n (%)	
	Older	Younger	Older	Younger	Older	Younger	Older	Younger	Older	Younger	Older	Younger
Zhao et al.	6.14 ± 2.57	5.26 ± 1.99	69 (13.09%)	37 (8.1%)	59 (11.20%)	77 (16.8%)	0.87 ± 0.52	1.23 ± 0.54	NA	NA	338 (64.14%)	197 (43.10%)
Liu et al.	5.28 ± 1.45	4.70 ± 1.65	2 (11.11%)	2 (5.26%)	3 (16.67%)	8 (21.05%)	NA	NA	18.89 ± 13.15	28.99 ± 7.03	9 (50.00%)	8 (21.05%)
Chen et al.	11.27 ± 18.04	7.60 ± 11.98	10 (18.18%)	4 (2.70%)	20 (36.36%)	59 (39.86%)	2.30 ± 4.57	7.00 ± 14.97	NA	NA	45 (81.82%)	72 (48.65%)
Lian et al.	5.03 ± 1.87	4.83 ± 1.56	9 (6.62%)	9 (1.38)	38 (27.94%)	196 (30.06%)	1.07 ± 0.52	1.23 ± 0.52	NA	NA	42 (30.88%)	92 (14.11%)

**Table:** (B) Inflammatory response and coagulation function of the aging patients with COVID-19.

Study	C-reactive protein (mg/L)		Interleukin-6 increased, n (%)		Procalcitonin increased, n (%)		Platelets (× 10 ^9^/L)		D-dimer increased, n (%)	
	Older	Younger	Older	Younger	Older	Younger	Older	Younger	Older	Younger
Zhao et al.	50.37 ± 55.31	21.13 ± 31.22	NA	NA	49 (9.30%)	13 (2.75%)	196.60 ± 84.14	213.00 ± 72.87	NA	NA
Liu et al.	23.63 ± 17.29	8.45 ± 12.59	NA	NA	NA	NA	217.67 ± 102.97	227.33 ± 82.43	NA	NA
Chen et al.	123.77 ± 214.68	111.33 ± 219.35	22 (40.00%)	22 (14.86%)	5 (9.09%)	2 (1.35%)	199.33 ± 298.42	234.00 ± 395.29	19 (34.55%)	7 (4.73%)
Lian et al.	23.10 ± 29.30	8.55 ± 11.07	NA	NA	NA	NA	169.67 ± 56.57	186.33 ± 52.75	NA	NA

When it comes to lymphocytes, the situation is opposite. The total number of lymphocytes in elderly patients is lower than that in young patients (SMD -0.47, 95% CI: -0.72 to -0.22, *p* < 0.01) (**Figure 4D**), and the risk of lymphocyte decline is higher than that in young patients (OR 2.73, 95% CI: 2.22–3.36, *p* < 0.01), and there is no significant heterogeneity (I^2^ = 0.00%) (**Figure 4E**). All these findings support the high inflammatory state in elderly patients because the total white blood cells increase in aging patients while the lymphocytes decrease inversely. The decline of lymphocytes also shows a fragile immune defense of the elderly population.

In terms of measuring the coagulation function, the platelet number of elderly patients is lower than that of the young group (SMD -0.22, 95% CI: -0.32 to -0.13, *p* < 0.01), and there is no significant heterogeneity (I^2^ = 0.00%) (**Figure 4F**). In terms of inflammation-related measures, the C-reactive protein (CRP) of elderly patients is higher than that of young people (SMD 0.65, 95% CI: 0.23–1.07, *p <*0.01) (**Figure 4G**) with a high heterogeneity (I^2^ = 91.92%) in a random-effects model, which also reflects the excessive inflammation in the elderly patients.

## Discussion

With the intensification of global aging, how to deal with the medical challenges brought about by the aging society is a topic of widespread concern. Aging is considered to be closely related to the increasing incidence of a series of chronic diseases (14, 15). Changes in the composition of multiple immune cells and the decline of immune function constitute “immune aging” in the natural aging process (16). Meanwhile, the global novel coronavirus epidemic reminds us that the prevention and control strategy of new infectious diseases in the elderly population is a more complex and comprehensive issue. Recent studies show that the incidence of COVID-19, the incidence of critical illness, and the mortality rate in the elderly population have increased significantly compared with the younger population (17). In addition, in past studies, the elderly population is also considered to have low responsiveness toward vaccine, which means that vaccines against COVID-19 may not provide adequate immune defense to the elderly population who need them most. However, there is still a lack of comprehensive systemic understanding of age-related changes in immunity. Moreover, the mechanism of how the aging immune disorders cause the incidence and mortality of COVID-19 increase is still unclear. What is more, age was always simply regarded as a confounding factor in many studies, which means that we have never treated age as a major factor to discuss its impact on emerging infectious diseases.

Here, we summarize three observational studies of new coronary pneumonia directly grouped by age and comprehensively analyze the true impact of age as a complex variable on COVID-19 disease. The main limitation of this meta-analysis is the limited number of studies included. Due to the small number of studies, we did not analyze the risk of bias either. What is more, the existing studies did not convey sufficient laboratory tests, such as myocardial enzymes, etc. In fact, we have found that various observational studies on COVID-19 do not contain a unified clinical symptom collection standard, which will bring challenges to subsequent systematic analysis of new diseases. At the same time, the laboratory examinations selected by different centers are not uniform, and some studies do not even list the normal range of indicators. Here, we propose that clinical manifestations and laboratory tests can be classified by different modules. In addition, our statistical analysis shows that, sometimes, the number of abnormal values of indicators is more attractive than direct statistics on numerical variation. Notably, we have also found several observational studies on elderly patients did not contain younger patients as controls (18, 19). Although some experiments have carried out a stratified analysis within the elderly patients (20), not all trials provide enough data on age. Therefore, our analysis is not yet able to do a systematic hierarchical analysis of elderly patients, which could be updated later.

However, we think that this preliminary summary analysis may provide a little help in specifying risk stratification according to age and establishing a multidisciplinary comprehensive treatment strategy model in this stage of the pandemic.

Our meta-analysis finds that the risk of hypertension, diabetes, and other cardiovascular diseases in elderly patients is significantly higher than that in young people. This means poor basic conditions of blood vessels and metabolic systems in te elderly patients, which might be related to the higher incidence of COVID-19, especially the critical illness, the poorer prognosis, and the higher mortality rate in the elderly population (21). This virus infects the human body through ACE2 receptors, which are widely expressed in the vascular endothelium (22). At the same time, this novel virus has also been detected in other organs of the human body (23). These evidences indicate that COVID-19 may be a systemic multiple organ damage disease (24). Poor vascular and metabolic conditions may also cause some elderly patients with COVID-19 to be more likely to have multiple organ damage, except the lungs, and increase the risk of death (25). In addition, this high risk also means that elderly patients need to add treatment for their basic chronic diseases as well as antiviral treatment because viral infection may, in turn, aggravate their own comorbidities. Therefore, the influence of preexisting conditions on the prognosis of infection is also crucial in understanding COVID-19. Recent studies show that smoking is likely to be related to the negative progress and adverse outcomes of COVID-19 (26), and smoking is proven to increase the gene expression of ACE2 (a protein that binds to SARS-CoV-2), which may promote COVID-19 infection (27). Similarly, there are many studies with a strong interest in basic comorbidities. Some studies initially discuss the diabetes management of patients with COVID-19 (28), and some studies from China discuss in detail the impact of comorbidities on the 2019-nCoV infection (29). It is revealed that patients with any comorbidities are worse than those without comorbidities in COVID-19. However, existing research did not pay special attention to the elderly. In this meta-analysis, our findings may further suggest that such management also needs to be treated differently according to age.

The elderly patients with basic chronic diseases need multidisciplinary standardized management. This means that more cardiovascular and metabolic disease doctors will be included in the MDT team to face the epidemic challenges jointly. The management of basic chronic diseases, such as hypertension, is often inseparable from ACEI/ARB drugs, which has attracted widespread attention in the prevention and control of new coronavirus epidemics (30). In fact, calcium antagonists are more widely used in elderly hypertensive patients for their better adaptability to relieve microvascular spasm and increase cerebral blood supply (31). This reminds us that dose adjustment and standardized use of calcium antagonists are a more important issue for the management of hypertension in elderly patients with COVID-19.

Meanwhile, we also analyzed the symptomatic differences between elderly patients and young infected persons. Although the data obtained are not enough, the going analysis suggests that there is no significant difference between these two groups. But this may not be a negligible negative result. Traditionally, it is believed that the elderly lack immunity, and their symptoms are severe after having a cold or flu, which might be a good sign for a doctor to screen. However, the initial features of elderly patients infected by 2019-nCov is relatively nonspecific, which is a new challenge for community screening in aging society.

In addition, changes of age-related molecules in the blood are considered as new entry points for understanding aging-related diseases (32). We also compared the blood routine and some serological indexes between the elderly patients and the young infected. The results suggest that elderly patients have more white blood cells and a higher risk of increased white blood cells, which can attribute the aging immune mobilization. The total number of lymphocytes in elderly patients is lower than that in young patients, and the risk of their lymphocyte decline is higher. Both results suggest immune aging and lack of defense against viruses. In addition, CRP, the inflammation-related index, has a higher risk in the elderly group, which might be related to the chronic inflammation caused by aging. This pro-inflammatory state may induce an acute exacerbation of older patients with COVID-19. All these results may lay a foundation for building an elderly targeted, anti-inflammatory therapy and interferon therapy to improve immunity.

In summary, our meta-analysis shows that elderly patients with COVID-19 have a higher risk of having chronic comorbidities, including hypertension, diabetes, and other cardiovascular diseases. At the same time, our results show that the clinical manifestations of elderly patients are not significantly different from those of younger patients. Their risk of fever is higher than that of younger patients, but their weakness is less severe than that of younger patients. This is obviously unexpected from what we thought in the past that the elderly would suffer severe symptoms once they became ill. Indifference in initial symptoms and subsequent high mortality make us boldly speculate that the indifference in symptoms of elderly patients with COVID-19 may cause a kind of confusion that they are not very serious, thereby delaying the treatment of the elderly. In addition, there is a certain significant difference between the blood routine and some serological indicators, which may be used as a guide for subsequent treatment selection and continuous monitoring. In addition, we look forward to studies in the future, including larger samples and longer-term follow-up, to evaluate the effect of age on the end-of-life events of 2019-nCov infection. Meanwhile, we encourage more cardiovascular and metabolic doctors to participate in the intensive treatment of elderly patients with COVID-19.

## Data Availability Statement

All datasets presented in this study are included in the article/supplementary material.

## Author Contributions

PJ and CY conceived the need for the article. CY drafted the initial version. PJ helped perform the final version with constructive discussions. PJ provided expert critical input regarding the content. All authors contributed to the article and approved the submitted version.

## Funding

This work was supported by grants from the National Science Fund for Distinguished Young Scholars (81625002), the National Natural Science Foundation of China (81930007, 81470389, 81500221, and 81800307), the Shanghai Outstanding Academic Leaders Program (18XD1402400), Innovative research team of high-level local universities in Shanghai, and Shanghai Municipal Education Commission Gaofeng Clinical Medicine Grant Support (20152209).

## Conflict of Interest

The authors declare that the research was conducted in the absence of any commercial or financial relationships that could be construed as a potential conflict of interest.
